# NIFTy: near-infrared fluorescence (NIRF) imaging to prevent postsurgical hypoparathyroidism (PoSH) after thyroid surgery—a phase II/III pragmatic, multicentre randomised controlled trial protocol in patients undergoing a total or completion thyroidectomy

**DOI:** 10.1136/bmjopen-2024-092422

**Published:** 2025-01-30

**Authors:** Julie Croft, Gemma Ainsworth, Neil Corrigan, Katie Gordon, Anna Perry, Maureen Twiddy, Mark Strachan, Jonathan Wadsley, Hisham Mehanna, Neil Sharma, Elizabeth Glenister, Deborah D Stocken, Sabapathy Prakash P Balasubramanian

**Affiliations:** 1 Clinical Trials Research Unit, Leeds Institute of Clinical Trials Research, University of Leeds, Leeds, UK; 2 Institute of Clinical and Applied Health Research, University of Hull, Hull, UK; 3 Consultant Endocrinologist, Metabolic Unit, Western General Hospital, NHS Lothian, Edinburgh, UK; 4 Weston Park Cancer Centre, Weston Park Hospital, Sheffield, UK; 5 InHANSE, Institute of Cancer and Genomic Sciences, University of Birmingham, Birmingham, UK; 6 University of Birmingham, Birmingham, UK; 7 Parathyroid UK, West Sussex, UK; 8 The University of Sheffield, Sheffield, UK; 9 Sheffield Teaching Hospitals NHS Foundation Trust, Sheffield, UK

**Keywords:** SURGERY, Clinical Trial, Thyroid disease

## Abstract

**Introduction:**

Postsurgical hypoparathyroidism (PoSH) is an iatrogenic condition that occurs as a complication of several different procedures with thyroid surgery being the most common. PoSH has significant short- and long-term morbidities. The volume of thyroid surgery is increasing, and PoSH is therefore likely to increase. Some studies have shown promising results using near-infrared fluorescence (NIRF) imaging in reducing the risk of PoSH which has the potential to significantly reduce morbidity and costs associated with monitoring and treatment.

**Methods and analysis:**

NIFTy is an unblinded, parallel group, multicentre, seamless phase II/III randomised controlled trial in patients undergoing total or completion thyroidectomy. The trial incorporates a process evaluation (IDEAL (Idea, Development, Exploration, Assessment and Long-term follow-up framework) 2a) to inform the trial protocol, a phase II (IDEAL 2b) analysis using a surrogate primary outcome of 1 day transient hypocalcaemia to determine early futility and phase III (IDEAL 3) assessment of the primary outcome of PoSH at 6 months after surgery. 454 participants will be randomised on a 1:1 basis to evaluate thyroid surgery with NIRF and indocyanine green against standard thyroid surgery in reducing PoSH at 6 months after surgery, with the phase II analysis occurring once data are available for 200 participants. Analysis in both phases will be using multilevel logistic regression incorporating random effects with respect to surgeon and adjusting for minimisation factors. Phase III secondary outcomes include protracted hypoparathyroidism, hypercalcaemia, complications, length of stay, readmissions and patient reported quality of life using the Short Form 36 Health Survey Questionnaire and Hypoparathyroid Patient Questionnaire instruments.

**Ethics and dissemination:**

NIFTy is funded by National Institute for Health and Care Research Efficacy and Mechanism Evaluation Programme (Grant Ref: 17/11/27) and approved by a Research Ethics Committee (reference: 21/WA/0375) and Health Research Authority (HRA). Trial results will be disseminated through conference presentations, peer-reviewed publication and through relevant patient groups.

**Trial registration number:**

ISRCTN59074092.

**STRENGTHS AND LIMITATIONS OF THIS STUDY**

- An embedded process evaluation was conducted during the trial set up, identifying and agreeing the key components of the intervention and informing planned data collection.
- NIFTy uses a seamless design that incorporates an early assessment of futility and full-scale evaluation of the near-infrared fluorescence with indocyanine green (NIRF+ICG) intervention within an efficient sample size.
- NIFTy aims to explore the mechanisms by which NIRF+ICG affects PoSH at 6 months after surgery and will provide high quality data on a range of post-surgical outcomes for total and completion thyroidectomy procedures.
- The trial allocation will be unblinded, though this limitation is mitigated by the objectivity of the phase II primary endpoint. Additional data on objective measures of calcium and active vitamin D supplement usage will be collected specifically to monitor potentially subjective components of the phase III endpoint.

## Introduction

 Postsurgical hypoparathyroidism (PoSH) is an iatrogenic condition that occurs as a complication of several different procedures. Thyroid surgery is the most common procedure associated with PoSH, and this is performed for a range of benign and malignant conditions (including multinodular goitre, hyperthyroidism, thyroid cancer). Over 12 000 thyroid operations are done annually in England alone (Hospital Episode Statistics (HES)), a significant proportion of which involves bilateral thyroid surgery, which carries an increased risk of this complication. The volume of thyroid surgery is increasing in the UK^1^ and worldwide, and so occurrence of PoSH is likely to increase. The prevalence of the problem is estimated to be around 22 per 100 000 in the Western world.^2 3^

PoSH has significant short- and long-term morbidity. In the short term, the condition can lead to troublesome symptoms including paraesthesia, spasms of extremities and larynx, anxiety, sensory disturbances, heart dysfunction and tetanic cramps. Its occurrence can prolong hospital stay^4^ and is associated with multiple hospital attendances, readmission to hospital,^5^ ongoing monitoring and treatment—all significantly increasing cost. In the long term, PoSH can cause a wide range of symptoms, a ‘high burden of illness’^6^ and a negative impact on different aspects of quality of life.^7^ Epidemiological studies have also shown that affected individuals have an increased risk of renal impairment, seizures, infections, depression and calcifications.^2 8^ A significant reduction in the incidence of PoSH would therefore have potential to significantly reduce morbidity and associated costs.

Although much research has focused on the incidence,^9^ prevalence^2 3 10^ and factors predisposing to this condition,^11^ reliable preventative measures have not been developed. Many alternative techniques have been explored in the literature. However, the effectiveness of these measures were summarised in a review^9^ showing that although temporary hypocalcaemia rates may be reduced, the impact on long-term PoSH is minimal.

Several novel intraoperative technologies have been investigated for their potential in identifying and preserving parathyroid glands during thyroid surgery, including near-infrared fluorescence (NIRF) imaging, which is based on either parathyroid autofluorescence or on the use of exogenous fluorophores such as indocyanine green (ICG).^12^ The potential for NIRF imaging in parathyroid preservation has been corroborated by other early phase studies^13^,^15^ and summarised in a recent review.^16^ Although promising, the results are considered preliminary and need to be validated in well-conducted, multicentre phase II and III studies. To address this gap, the NIFTy trial will investigate the use of near-infrared imaging devices, specifically designed, and CE (Conformité Européenne) marked, for thyroid and parathyroid surgery in a phase II/III setting.

The Medical Research Council guidance on the development of complex interventions states that the randomised controlled trials (RCTs) should standardise the content and delivery of the intervention under investigation. In surgical trials, it is important to establish standards for both the standard and ‘novel’ procedure to ensure interventions are delivered as intended, and so outcomes can be attributed to the intervention. Therefore, an embedded process evaluation was conducted during the trial set up to identify and agreed the key components of the intervention and determine which elements of the intervention should be mandated and what should be optional.

## Methods and analysis

### Trial design

This is an unblinded, parallel group, multicentre, seamless phase II/III (IDEAL (Idea, Development, Exploration, Assessment and Long-term follow-up framework) stages 2a–3), RCT comparing thyroidectomy surgery with NIRF+ICG against standard thyroidectomy surgery to determine the effect on PoSH at 6 months in patients undergoing thyroidectomy.

The trial is designed as a phase III trial with an interim analysis (termed hereafter as the phase II analysis) to allow the trial to stop early for futility.^17 18^ Recruitment will continue while the phase II analysis is ongoing. The trial will continue beyond the phase II analysis only if there is sufficient evidence of superiority of NIRF+ICG when compared with the control; otherwise, the study will stop, concluding futility. If the trial proceeds to the final phase III analysis, recruitment will continue until the full sample size of 454 patients (227 per treatment group) is reached. In both phases, participants will be individually randomised to receive either standard thyroid surgery or thyroid surgery using NIRF+ICG on a 1:1 basis using minimisation (with a random element).

NIFTy aims to determine the efficacy of NIRF imaging (using parathyroid autofluorescence and with ICG) in reducing the risk of PoSH after bilateral thyroid surgery in a multicentre RCT comparing NIRF imaging versus standard surgery.

#### Phase II objective

The primary objective of the phase II component is to determine whether to continue to recruit to the phase III component of the trial. This decision will be based on early assessments of efficacy measured in terms of 1-day postoperative hypocalcaemia.

#### Phase III objectives

The primary objective of the phase III trial is to investigate the efficacy and mechanism of NIRF+ICG during thyroidectomy in reducing the rate of PoSH compared with standard thyroidectomy at 6 months postsurgery.

Secondary objectives are to compare postoperative parathyroid hormone (PTH) levels, incidence of transient hypocalcaemia, incidence of protracted hypoparathyroidism, intra- and postoperative complications, length of stay in hospital after surgery, patient quality of life, readmission to hospital and incidence of hypercalcaemia between the study arms. NIFTy will also explore the mechanism of the intervention by investigating the effects of key surgical components identified in the process evaluation.

### Process evaluation

Prior to development of the NIFTy trial protocol, a process evaluation took place. The aim of the process evaluation was to identify and agree the key components of a total and completion thyroidectomy, including how NIRF+ICG should be used in the NIFTy RCT. The process evaluation was informed by a qualitative case study approach developed by Blencowe and colleagues^19^ and used to develop a trial surgical protocol and associated reporting mechanisms. The work consisted of three stages: (1) identification of current clinical practice and a mapping of the surgical steps; (2) survey to understand wider clinical practice; and (3) consensus meeting to agree which steps of the total/completion thyroidectomy should be mandatory/optional, determine the list of data items to be recorded and agree the ways in which NIRF+ICG should be used within the trial.

#### Stage 1

A non-participant observational case study planned to recruit a purposive sample of ~15 total and completion thyroidectomies from three National Health Service (NHS) Trusts. Selected cases encompassed different degrees of difficulty and surgeries undertaken by different surgeons to map variation in practice. Two researchers (one clinically qualified) observed each surgery to map clinical practice using a surgical checklist and video recordings from first incision to final sutures of each procedure. Postoperative interviews with the lead surgeon were conducted immediately following the surgery, where possible, or within 24 hours. Semistructured interviews (see online supplemental file 1) allowed the exploration of surgeons’ thoughts about how the operation went, changes to planned approach and use of the NIRF in practice (with and without ICG). Data were analysed in parallel with data collection. Surgical steps and interview data were charted for analysis by a clinical researcher to enable comparison of operating technique adopted by each surgeon, usual practice and variations in approach.

#### Stage 2

Online surveys were developed in conjunction with the clinical team. The first (see online supplemental file 2) assessed current practice: of identifying and determining viability of the parathyroid glands during surgery and current use of NIRF; the second (see online supplemental file 3) presented the surgical steps from stage 1 to determine the peri- and intraoperative steps used by clinicians across the UK. Respondents were asked to consider (a) which surgical steps they believe are (un)related to the preservation of the parathyroid glands and which should be mandated/optional in the NIFTy trial and (b) confirm the acceptability of using NIRF+ICG to aid identification of the parathyroid glands. Results were analysed descriptively (median/range), and content analysis was used to analyse free-text questions.

#### Stage 3

An online consensus meeting was used to agree the surgical protocol and final list of items to be collected (items believed to influence outcomes). Members were selected using a key informant approach on the basis of their experience and expertise in thyroid surgery, use of NIRF and research experience in the area. The meeting was chaired by an independent clinician with a strong track record of research in the area. Stages 1 and 2 findings were presented for consideration by the panel. Members were asked to agree (a) the mandatory/optional aspects of the intervention; (b) steps to be taken to preserve the parathyroid glands; (c) the use of other surgical interventions (eg, intraoperative recurrent laryngeal nerve monitoring); (d) timing of autofluorescence and use of ICG during the trial; and (e) the level of flexibility surgeons should be allowed. The output was a surgical manual for use in the trial and material to inform the development of surgical data collection.

### Trial setting and recruitment

Participants will be recruited from approximately 10 research sites in the UK over a 32-month period. A phase II interim analysis will be performed after adequate phase II primary endpoint data (transient hypocalcaemia on the day after surgery) has been collected, approximately 1 month after randomising 200 participants.

Patients requiring total or completion thyroidectomy will be identified prior to elective surgical admission. Suitability for trial inclusion will be assessed according to the eligibility criteria in table 1, and potential participants will be approached in the surgical clinic.

**Table 1:** NIFTy patient eligibility criteria

Inclusion criteria	Exclusion criteria
Aged ≥18 years. Able to provide written informed consent. Due to undergo total or completion thyroidectomy with or without central neck dissection (indications for surgery may include Graves’ disease, suspected or confirmed thyroid cancer and goitre with compressive effects). ASA (American Society of Anesthesiologists) </= 3. Able and willing to comply with the terms of the protocol including participant completed questionnaires.	Patients undergoing concomitant parathyroid or thoracic surgery. Patients undergoing reoperative thyroid surgery (except completion thyroidectomy after previous contralateral lobectomy). Documented hypo or hypercalcaemia within the last 3 months prior to planned date of thyroid surgery, defined as adjusted calcium <2.1 mmol/L or >2.6 mmol/L. Pregnancy. Known allergy to ICG, iodine or iodine dyes. Patients with significant renal impairment (defined as eGFR <40 mL/min/1.73 m^2^ (or a serum creatinine value >10% of upper value for normal institutional limits if eGFR is not performed locally). Taken calcium or active vitamin D supplements (such as alfacalcidol or calcitrol) in the 2 weeks prior to randomisation or due to receive prophylactic treatment with calcium and/or active vitamin D supplements perioperatively.

eGFRestimated glomerular filtration rateICGindocyanine green

A verbal explanation of the trial along with the approved patient information sheet/informed consent form (PIS/ICF) (see online supplemental file 4) will be provided by a suitably qualified member of the healthcare team for the patient to consider. Patients may also be identified from surgical waiting lists after attending for their preoperative assessment. Patients identified in this way will be contacted by letter, including a copy of the PIS, and will have the opportunity to discuss the trial during a phone call with the surgeon.

The PIS will provide detailed information about the rationale, design and personal implications of the trial, and patients will be given the opportunity to discuss the trial being invited to provide informed, written consent for their participation. Patients will be given as much time as possible to consider their participation in the trial and ideally a minimum of 24 hours. The right of the patient to refuse participation without giving reasons will be respected. Informed written consent will be obtained by the principal investigator (PI) or appropriate, delegated, healthcare professional as detailed on the authorised personnel log, in accordance with the principles of Good Clinical Practice and Declaration of Helsinki 1996, prior to being randomised.

Participants will remain free to withdraw from the trial at any time without giving reasons and without prejudicing any further treatment. Loss of mental capacity of a participant after giving informed consent for the trial is expected to be a rare occurrence, and no further trial procedures or data collection will occur from this point. Any data collected up to the point of withdrawal will be kept on record and used in the trial analysis.

### Eligibility

*Research site eligibility*. Participation of research sites will be dependent on their ability to perform thyroid surgery using NIRF technology using a single device capable of detecting both parathyroid autofluorescence and ICG fluorescence and the predicted capability to recruit a minimum of 12 patients per year into the NIFTy trial.

*Surgeon eligibility* Prior to randomising participants, all participating surgeons must have observed at least one thyroid/parathyroid operation performed using NIRF+ICG, performed a minimum of five thyroid/parathyroid operations using NIRF+ICG and performed a minimum of 20 thyroid operations per year.

Participant eligibility criteria are detailed in table 1.

### Randomisation and blinding

Following confirmation of written informed consent and eligibility, participants will be randomised into the trial. Randomisation will be performed centrally using the central automated 24-hour randomisation system. Participants will be randomised on a 1:1 basis to receive thyroid surgery performed either as per standard care or with NIRF+ICG and allocated a unique trial number. A computer-generated minimisation programme that incorporates a random element will be used to ensure treatment groups are well-balanced for the following participant characteristics: operating surgeon, indication (Graves’ disease, cancer, etc) and sex. Clinical assessments and baseline questionnaires will be completed before randomisation. Treatment allocation will not be blinded to participants, medical staff or clinical trial staff.

The timing of clinical assessments and data collections points are summarised in table 2.

**Table 2:** Clinical assessments and data collection timepoints

Events	Baseline/preop	Surgery	1 day post-op	1 month post-op	6 months post-op
Clinical assessments/investigations					
Clinical examination	**✓**		**✓**	**✓**	**✓**
Biochemistry	**✓**		**✓**	**✓**	**✓**
Trial consent	**✓**				
Operative details		**✓**			
Complications		**✓**		**✓**	**✓**
Data collection time points					
Eligibility eCRF	**✓**				
Randomisation eCRF	**✓**				
Baseline eCRF	**✓**				
Operative eCRF		**✓**			
1 day after surgery f/up eCRF			**✓**		
1 month after surgery f/up eCRF				**✓**	
6 months after surgery f/up eCRF					**✓**
HRQoL					
SF-36	**✓**			**✓**	**✓**
HPQ-28	**✓**			**✓**	**✓**

eCRFelectronic case report formHPQ-28Hypoparathyroid Patient QuestionnaireHRQoLhealth related quality of lifeSF36Short Form 36 Health Survey Questionnaire

Preoperative investigations and preparation will be as per institutional protocol. A biochemical assessment will be carried out including thyroid function, renal function, calcium, PTH, vitamin D, magnesium, phosphate, and urea and electrolytes, details of which will be recorded on the trial case report forms (CRFs). Participants must not be given prophylactic treatment with calcium and/or active vitamin D supplements perioperatively. However, any vitamin D deficiency that is detected during investigation may be treated as per local protocols.

For participants randomised to the surgery without NIRF imaging (standard care) arm, the total or completion thyroidectomy, with or without central neck dissection, should be performed as part of standard care. For participants randomised to the surgery with NIRF+ICG arm, the total or completion thyroidectomy, with or without central neck dissection, will be performed using NIRF imaging with and without ICG during surgery. The use of NIRF imaging will be in accordance with the NIFTy surgical manual developed in the process evaluation. The NIFTy surgical manual describes the components/steps of thyroid surgery that may impact the risk and severity of PoSH and provides guidance to participating surgeons on how these steps should be carried out for trial participants (regardless of the treatment arm), as well as which components are mandatory, strongly recommended or optional.

**Figure 1:**
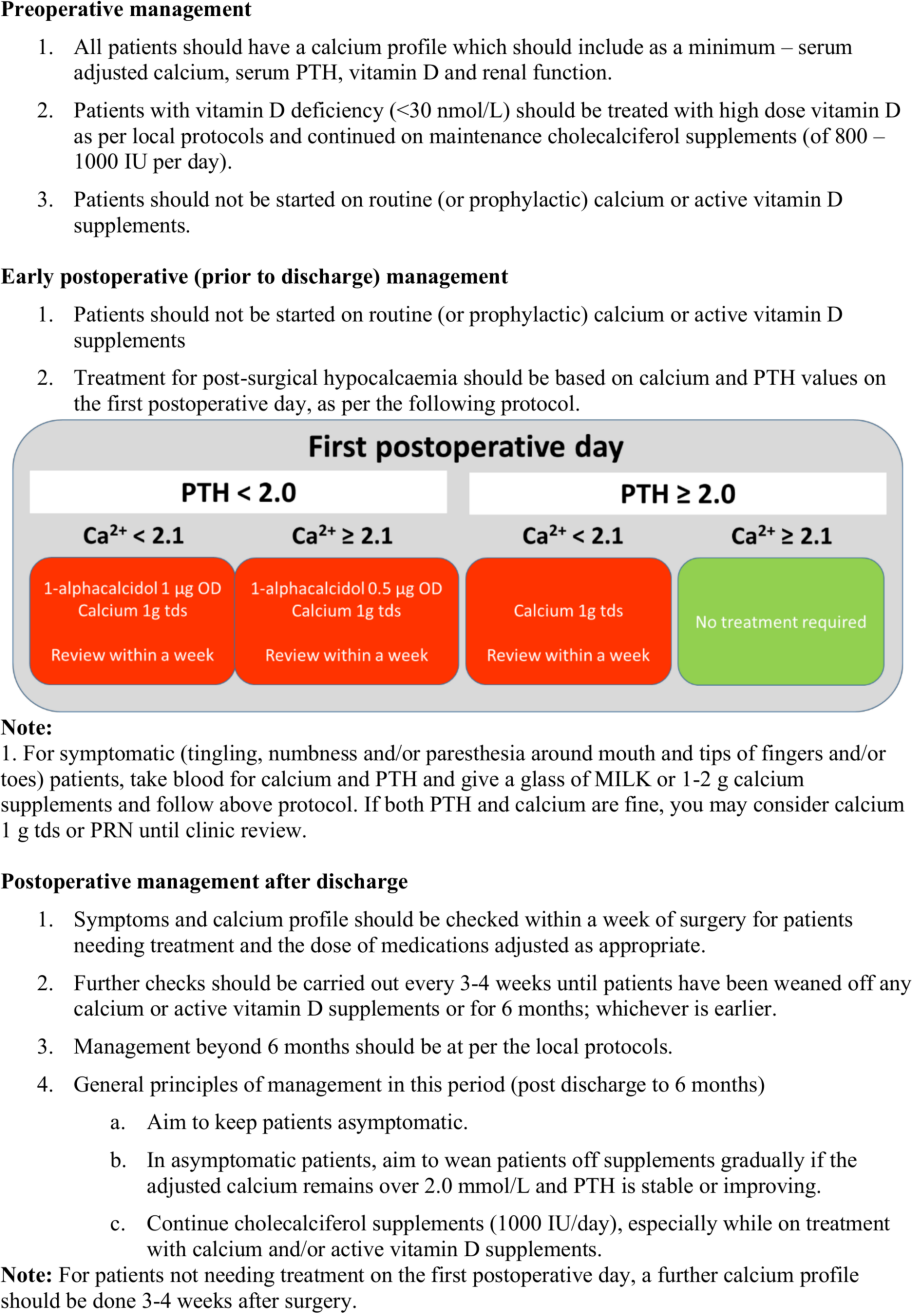
Protocol for assessment and treatment of postsurgical hypocalcaemia and hypoparathyroidism for patients enrolled in the NIFTy trial. PRN, pro re nata (as required); OD, optical density; PTH, parathyroid hormone; tds, three times a day.

Postoperative care will be in accordance with local and national protocols and guidance and will include an evaluation for hypocalcaemia/hypoparathyroidism by review of symptoms and biochemistry. The detection and treatment of acute post-thyroidectomy hypocalcaemia will be performed as per local protocols; however, prophylactic calcium supplements must not be given to patients before assessment of calcium levels on the day after surgery, as this can affect the validity of the 1-day endpoint. A protocol developed and validated in the Sheffield unit may be used as a guide for participating sites if required (see figure 1: protocol for assessment and treatment of post-surgical hypocalcaemia and hypoparathyroidism). Most patients will be discharged 1–2 days after their surgery. Participants will be reviewed at: 1 day after surgery (inpatient), 1 month postsurgery (outpatient clinic) and 6 months postsurgery. Any further visits will be according to local clinical practice.

Participants will complete health-related quality of life questionnaires at baseline and at 1 and 6 months post-surgery.

In line with usual clinical care, cessation or alteration of treatment at any time will be at the discretion of the attending clinician or the participant themselves.

### Data collection

Clinical data will be collected at baseline, operation, and at 1 day, 1 month and 6 months postsurgery; participant-completed data will be collected at baseline and at 1 and 6 months postsurgery. Baseline participant-completed questionnaires will be collected on paper, and follow-up participant-completed questionnaires will be collected either on paper or electronically via patient-reported outcome software, dependent on participant choice. All other data collection will be via paper CRFs or via remote data entry on electronic CRFs managed by the Clinical Trials Research Unit (CTRU) at the University of Leeds.

Data will be monitored for quality and completeness by CTRU. Missing data will be requested from sites until received and confirmed as unavailable or trial analysis begins. The sponsor reserves the right to conduct source data verification to monitor trial integrity.

Information collected during the trial will be kept strictly confidential. Information will be held securely on paper and electronically at Leeds CTRU who will comply with all aspects of the Data Protection Act 2018. If a participant withdraws consent from further trial treatment and/or further data collection, data up to the point of withdrawal will remain on file and included in the analysis.

### Outcome measures

#### Phase II primary outcome measure

Transient hypocalcaemia, defined as any adjusted calcium of <2.1 mmol/L at 1 day postsurgery.^20^

#### Phase III primary outcome measures

Incidence of PoSH at 6 months postsurgery—defined as the need for calcium and/or vitamin D supplements to treat symptoms or maintain adjusted calcium in the low normal range (between 2.1 and 2.3 mmol/L). This is in accordance with the national British Association of Endocrine and Thyroid Surgeons definition.^21^

#### Phase III key secondary outcome

Postoperative PTH over 6 months, measured at 1 day, 1 month and 6 months postsurgery.

#### Phase III secondary outcome measures

To compare NIRF+ICG and standard care in terms of:

- Transient hypocalcaemia.
- Protracted hypoparathyroidism, defined as either an adjusted calcium of <2.1 mmol/L or the need for calcium and/or vitamin D supplements to treat symptoms or maintain adjusted calcium in the low normal range at 1 month post-surgery.
- Intra-operative complications graded using the ClassIntra classification.^22^
- Postoperative complications within 6 months of operation graded using the Clavien-Dindo classification.^23^
- Length of hospital stay in days post-surgery.
- Health related quality of life (HRQoL) at baseline, 1–6 months postoperation using the Short Form 36 Health Survey Questionnaire and Hypoparathyroid Patient Questionnaire instruments.
- Any episode of readmission to hospital within 6 months of operation
- Hypercalcaemia occurring within 6 months of operation, defined as any adjusted calcium >2.6 mmol/L measured at 1 day, 1 month and 6 months post-surgery.

#### Phase III mechanistic outcome measures

To explore the effect of treatment-related decision-making on patient outcomes.

### Statistical considerations and analyses

#### Sample size

**Figure 2:**
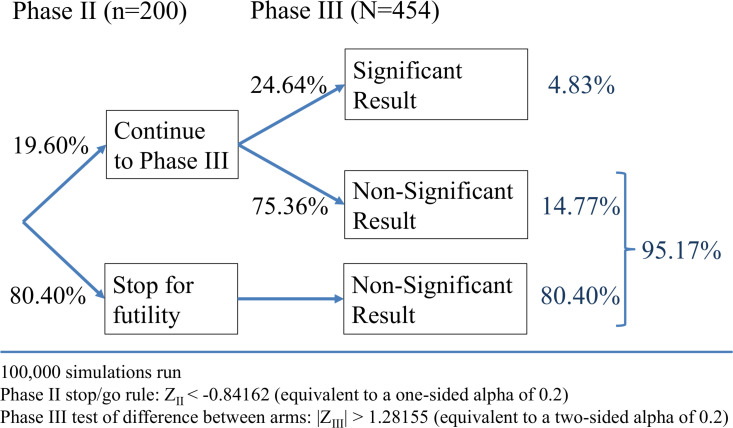
Operating characteristics of the proposed design under the null hypothesis.

Bayesian optimisation^24 25^ was used to determine the combination of total sample size, phase II sample size and the decision boundaries for the test statistics at the phase II and phase III analyses (Z_II_ and Z_III_ in figures2  3) that minimise the expected sample size required while maintaining an overall significance level of 5% and overall power of at least 80%. Each design explored during the optimisation process was estimated empirically via simulations, using 1000 repeats, and the operating characteristics of this chosen optimal design were re-evaluated using 100 000 repeats. The chosen design has an overall type I error rate of 4.83% and achieves an overall power of 89.33%.

**Figure 3:**
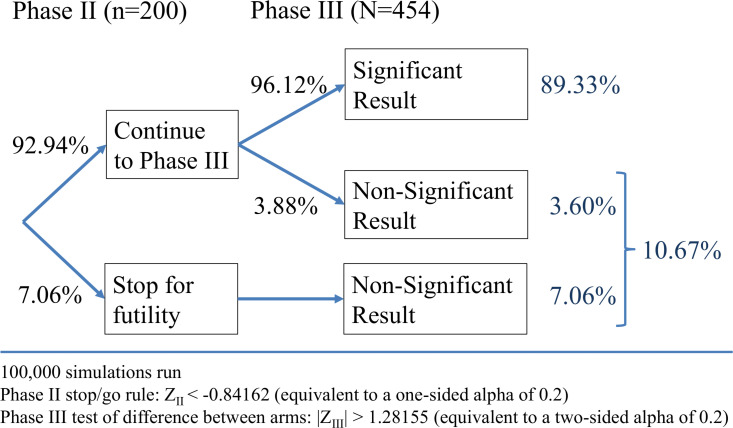
Operating characteristics of the proposed design under the alternative hypothesis.

The target sample size is 454 patients (227 per treatment group), with an interim phase II analysis after 200 patients (100 per treatment group), to allow the trial to stop early due to futility. Assuming a 6-month PoSH rate of 10% in the control group, and 1-day hypocalcaemia rate of 25% and 12.5% in the control and the NIRF+ICG groups, respectively, this design yields around 89% power at the 5% level of significance to detect a relative reduction in 6-month PoSH of at least 70% (ie, a reduction to 3% 6-month PoSH in the NIRF+ICG group), allowing for a 10% drop-out rate. Additionally, this sample size estimate relies on the key assumption that patients who are not hypocalcaemic at 1 day postsurgery will not experience PoSH at 6 months.

#### Analysis methods

All analyses and patient populations will be predefined within the trial’s statistical analysis plan and reporting will be in line with Consolidated Standards of Reporting Trials guidelines. The primary phase II and phase III analyses will be conducted using the principles of intention-to-treat (ITT), meaning that participants will be analysed in the group to which they were randomised irrespective of whether or not they receive their allocated intervention. Patterns of missing data will be explored, and analyses appropriate to the suspected missing data mechanism will be conducted, where necessary.

#### Interim analysis (phase II analysis)

Only the phase II primary endpoint will be assessed at the phase II analysis.

The phase II analysis will compare the 1-day hypocalcaemia rates between the arms using multilevel logistic regression, incorporating random effects with respect to surgeon and adjusting for the minimisation factors. At the phase II analysis, the trial will continue only if there is sufficient evidence of superiority of the experimental treatment compared with the control. This will be determined via a one-sided critical region for Z_II_, the Z-value from the multilevel logistic regression model’s Wald test of the adjusted log-odds ratio of 1-day hypocalcaemia. If Z_II_<−0.84162, then the trial will continue; otherwise, it will stop for futility. This decision boundary is equivalent to testing using a one-sided 20% significance level at the phase II analysis. The trial is designed based on a binding stop/go decision.

#### Final analyses (phase III analyses)

The primary phase III analysis will compare 6-month PoSH rates between the arms using multilevel logistic regression incorporating random effects with respect to the surgeon and adjusting for the minimisation factors. At the phase III analysis, evidence of a difference between the treatment groups will be determined via a two-sided critical region for Z_III_, the Z-value from the multilevel logistic regression model’s Wald test of the adjusted log-odds ratio of the 6-month PoSH. If |Z_III_|>1.28155, then the trial will conclude that there is a significant difference between the treatment arms. This decision boundary is equivalent to testing using a two-sided 20% significance level.

Secondary endpoints with binary measures will be analysed using multilevel logistic regression adjusting for the minimisation factors, incorporating random effects with respect to surgeon. Secondary endpoints with continuous measures will be analysed using multilevel generalised linear models incorporating random effects with respect to surgeon and assuming normal errors at the patient level. If the assumption of normal errors is clearly violated by the observed response data, then transformations of the response variable as well as alternative distributional assumptions will be explored, and the choice of a transformation and/or alternative distribution will be driven by comparative measures of model fit. Models for endpoints that are measured at multiple time points will also include an additional level to account for repeated measures, so that longitudinal effects can be assessed.

The mechanism of the NIRF+ICG intervention and how treatment-related decision-making is affected will be investigated using exploratory methods looking at the effects of the key surgical components identified in the embedded process evaluation.

### Monitoring

An independent data monitoring and ethics committee (DMEC) will annually review patient safety outcomes and data quality and monitor underlying assumptions of the statistical design. The DMEC, in the light of the interim data, will advise the Trial Steering Committee (TSC) if there is justification to consider closing the trial, including in the event that the results of the phase II analysis (interim analysis) demonstrate proof beyond reasonable doubt that the trial should be shopped in accordance with the planned stopping rule.

Information on complications will be collected from randomisation to 6 months postsurgery. Serious complications will be subjected to expedited reporting where sites will inform CTRU within 24 hours of becoming aware. Suspected or confirmed pregnancies and all deaths from randomisation until 6 months postsurgery will be reported to CTRU.

### Patient

The grant application was reviewed by two national patient charities and changes made to the outcomes as a result of these reviews. A patient and public involvement (PPI) representative was involved in the development of the grant application and therefore during the design of the research to input into the patient acceptability of the trial processes. The PPI representative has also reviewed the content of trial documents for patients, for example, the PIS/ICF document, questionnaire cover sheets and patient letters. There will be two PPI representatives involved in management of the research, one through the Trial Management Group (TMG) and one through the TSC. Views of the PPI representatives will be sought throughout the trial, and the PPI members will be closely involved in the dissemination of findings at the end of the trial.

### Ethics and dissemination

NIFTy is sponsored by the Sheffield Teaching Hospitals NHS Foundation Trust, and ethical approval was obtained from Wales Research Ethics Committee 5 Bangor (reference: 21/WA/0375) and Health Research Authority (HRA). All amendments will be submitted for approval and communicated to sites in accordance with HRA guidelines. The CTRU at the University of Leeds will have responsibility for the coordination of the trial.

Trial supervision will be established according to the principles of GCP and in line with the NHS UK Policy Framework for Health and Social Care. This will include establishment of a core Project Team, TMG, an independent TSC and independent DMEC.

The trial is registered with the ISRCTN registry (59074092). Trial results will be disseminated at relevant clinical conferences and societies, published in peer-reviewed journals and disseminated through relevant patient groups. Authorship will be according to International Committee of Medical Journal Editors guidelines. Data will be made available at the end of the trial once all key analyses are complete.

## Discussion

Technology in surgery is often incorporated into clinical practice in the absence of good quality evidence to support its effectiveness. RCTs are few and only a minority of surgical interventions have traditionally been based on RCT evidence.^26^,^28^ The challenges in detailed and comprehensive evaluation of surgical techniques and technologies have been well documented.^29^ The need to evaluate surgical technology prior to its introduction into routine clinical practice is now recognised, and this is now encouraged and facilitated by the introduction of the IDEAL framework.^30 31^

Several technologies and surgical approaches have been evaluated for their effectiveness in reducing the risk of PoSH after thyroid surgery. The focus of such interventions has been to help the surgeon identify and preserve the viability of parathyroid glands during the resection of the thyroid. Following initial reports of parathyroid fluorescence at certain wavelengths of light without exogenous fluorophores^32^,^34^ and reports of the fluorescent guiding potential of some chemicals already in clinical use (such as methylene blue and ICG),^35^ a number of case reports and series have demonstrated the potential utility of fluorescent guided identification and preservation of parathyroid glands during thyroidectomy.^36^ Some randomised trials have now demonstrated that use of autofluorescence and ICG-based fluorescence reduce the number of inadvertently resected parathyroid glands, increase postsurgical PTH levels, reduce postsurgical hypocalcaemia rates and the need for treatment; however, the impact on long term PoSH has not been convincingly demonstrated.^37 38^

NIFTy is the first UK multicentre trial in thyroid surgery. The trial aims to both assess the mechanisms by which fluorescent imaging reduces PoSH and to pragmatically evaluate the effectiveness of the technology in a range of centres and across a number of different thyroid surgeons.

## supplementary material

10.1136/bmjopen-2024-092422 online supplemental file 1

10.1136/bmjopen-2024-092422 online supplemental file 2

10.1136/bmjopen-2024-092422 online supplemental file 3

10.1136/bmjopen-2024-092422 online supplemental file 4
