# Supplementary material for: NIFTy: near-infrared fluorescence (NIRF) imaging to prevent postsurgical hypoparathyroidism (PoSH) after thyroid surgery—a phase II/III pragmatic, multicentre randomised controlled trial protocol in patients undergoing a total or completion thyroidectomy
Source: BMJ Open. 2025 Jan 30;15(1):e092422. doi: 10.1136/bmjopen-2024-092422 (PMC11784174; doi:10.1136/bmjopen-2024-092422)
Supplement: online supplemental file 1 [file bmjopen-15-1-s001.docx]

**SUPPLEMENT 2: NIFTy Process Evaluation Surgeon Interview (Follow up interview)**

**Topic guide**

**[depending on time between surgeries, if may be possible to ask about several patients – if so, adapt topic guide appropriately. If interview is about one patient, remember that some questions will not need to be asked for subsequent interviews with that surgeon].**

Repeat introduction and thank the surgeon for participating, reinforce confidentiality.

As discussed, we are trying to understand the complexity of surgical interventions within trials. We are interested in your views on the intervention and the factors that you consider important when performing a completion or total thyroidectomy so we can develop a protocol that the surgical community can accept.

Questions

1. Can you talk me through a routine [completion/total thyroidectomy]? [only needed for first interview – or if undertaking one interview to discuss all surgeries]
2. What do you think are the most important elements/steps of the operation in relation to identification of the parathyroid glands and their preservation? How important are each of these elements/steps to reduce the risk of PoSH?
3. Do you make planned modifications for particular patients?
   Probe: What do you think constitutes an easy or difficult patient?
    In which case and why?
   Do you think that varying the procedure has any implications for the risk of PoSH?(e.g. was the modification to reduce the risk?)
4. What are the main factors influencing how easy or difficult the operation is? (patient related? (e.g retrosternal extension, central neck dissection, size of thyroid gland, presence of Grave’s disease) Equipment?
5. Thinking specifically about patient X operation. In this surgery you used NIRF at [XXXXXX] point(s). What influenced your decision to use the fluorescence (auto fluorescence or dye) at these points? (e.g. surgical expertise, size/ location of glands, pathology)
   Prompt – ask about each separately if both were utilised and if dye was used on multiple occasions. [if discussing more than one patient repeat question for subsequent patients]
6. What do you think about the timing of the use of (auto fluorescence or dye) fluorescence? When else might it have been useful to use it?
7. When looking at auto fluorescence, and dye fluorescence, what are you looking for in the surgical field? How do each differ in terms of how useful they are to influence your actions? What knowledge would you want to pass on to other surgeons?
8. Do you think that the procedure can be standardised? If so, should it be standardised within the trial? (ask about standardising operation steps and standardising when and how often to use NIFR and dye)
   Why do you think it is a good or bad idea
   Standardisation between or within centres
   Standardisation of equipment or just steps in procedure
   Any barriers to standardisation?
9. Do you think other elements/components of the surgery (other than the use of the NIFR and dye) should be performed **the same** in all centres participating in the NIFTy trial? Can they be standardised?
   1. Appropriate positioning of patient – neck extended and stabilised
   2. Parts painted and draped
   3. Incision, subplatysmal flaps, separation of strap muscles in midline
   4. Lobectomy (one followed by another)
      1. Separation of lobe from strap muscles
      2. Opening of planes down to prevertebral fascia (not all do this step)
      3. Upper pole capsular dissection and mobilisation, looking for superior glands
      4. Identification of RLN (if possible) at this stage
      5. Look for inferior glands
      6. Continue capsular dissection (aiming to avoid devascularisation of glands)
      7. Complete lobectomy
   5. Look at parathyroids (if seen previously) – devascularised or not? If not viable, autotransplant
   6. Secure hemostasis
   7. Wound closure
10. What did you feel about using the decision recording tool intra-operatively? What refinements do you think it needs? What could the decisions be? What options should be available to select?
11. What did you like or dislike about using the device and fluorescence?
12. What are your feelings about being observed and/or videoed in the operating theatre? Did this influence performance or behaviour in any way, or not? [if discussing multiple surgeries rephrase to ask if this differed between surgeries]
13. END

**Ask for sociodemographic details, grade and experience (i.e number of previous cases performed). Thank the surgeon again and re-iterate confidentiality**
