# Supplementary material for: NIFTy: near-infrared fluorescence (NIRF) imaging to prevent postsurgical hypoparathyroidism (PoSH) after thyroid surgery—a phase II/III pragmatic, multicentre randomised controlled trial protocol in patients undergoing a total or completion thyroidectomy
Source: BMJ Open. 2025 Jan 30;15(1):e092422. doi: 10.1136/bmjopen-2024-092422 (PMC11784174; doi:10.1136/bmjopen-2024-092422)
Supplement: online supplemental file 2 [file bmjopen-15-1-s002.docx]

**SUPPLEMENT 3: NIFTy Process Evaluation Survey 1**

1. Which hospital do you work at? Open text
2. Please provide your email address if you are happy to be contacted again in relation to use of fluorescent imaging in thyroid surgery (optional)
3. How many years have you worked as a consultant? 0-5/ 6-10/10+
4. Approximately how many thyroid operations (of any kind) do you perform per year? Please use pre-covid figures if appropriate. (free text)
5. When performing thyroid surgery, do you look for parathyroid glands? Always/ usually/ sometimes/ rarely/ never
6. If you look for parathyroid glands during surgery, could you indicate at what stage you look for them? during dissection of upper pole/ during lower pole dissection/ during lateral dissection of gland/ not applicable
7. Do you evaluate the specimen after excision to see if there may be parathyroid glands attached to it? Always/ sometimes/ never/ not sure.
8. Do you assess the viability of the parathyroid glands that you have identified? yes/no/sometimes
9. To assess viability of parathyroid glands, which of the following features have you made use of? Appearance/ bleeding on incision/ look for feeding vessel (tick all that apply)
10. If parathyroid glands are not viable (in your opinion) do you auto-transplant? Yes/ no/ maybe
11. If you do auto-transplant, please mention the site of auto-transplantation (open text)
12. Please describe any tips or techniques you consider to be important in parathyroid gland preservation. Open text
13. Do you use any intraoperative adjuncts /technology to aid in parathyroid preservation during thyroid surgery? (e.g. methylene blue, autofluorescence, ICG based fluorescence, other: please state)
14. Do you have access to fluorescent imaging for use in thyroid surgery? (open text)
15. Please indicate your specialty (open text)
16. If applicable, please can you describe the produce/device you have access to, or plan to obtain (open text)
17. Would you be interested in using autofluorescence and ICG based fluorescence during thyroid surgery to aid preservation of parathyroid glands? (Y/N)
