# Supplementary material for: NIFTy: near-infrared fluorescence (NIRF) imaging to prevent postsurgical hypoparathyroidism (PoSH) after thyroid surgery—a phase II/III pragmatic, multicentre randomised controlled trial protocol in patients undergoing a total or completion thyroidectomy
Source: BMJ Open. 2025 Jan 30;15(1):e092422. doi: 10.1136/bmjopen-2024-092422 (PMC11784174; doi:10.1136/bmjopen-2024-092422)
Supplement: online supplemental file 4 [file bmjopen-15-1-s004.docx]

**Delete this line, then print first page of Information Sheet and Consent Form on Trust/Hospital headed paper**

**
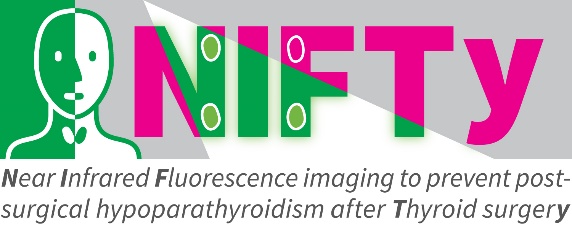
**

**NIFTy: N**ear **I**nfrared **F**luorescence (NIRF) Imaging to prevent Post-surgical Hypoparathyroidism (PoSH) after **T**hyroid Surger**y** (NIFTy) - A phase II/III pragmatic, multicentre randomised controlled trial

PARTICIPANT INFORMATION SHEET AND INFORMED CONSENT DOCUMENT

A large-print version of this sheet is available on request

You have been invited to take part in a research study called “NIFTy”

Before you decide if you want to take part, we would like to explain why the research is being done, how we will use the information we have about you, and what the research study will involve.

Please read this information carefully and discuss it with others if you like. Ask us if anything is unclear, or if you would like more information.

**Once you have read this information, your doctor or nurse will talk to you about the study again and you can ask further questions.**

- Part 1 tells you the purpose of this study and what will happen to you if you take part.
- Part 2 gives you more detailed information about the conduct of the study.
- Part 3 gives some additional information about how your information will be used.

Please take time to decide whether or not you wish to take part.

**How to contact us**

If you have any questions about this study, please talk to your doctor at

<<Enter PI, nurse name >>

<< Contact details for site>>

**Thank you for reading this information sheet.**

**Part 1 - overview**

What is the purpose of this study?

The NIFTy study is for participants who have a condition of the thyroid gland, such as Graves’ disease, goitre or thyroid cancer, and need to have an operation to remove all of their thyroid gland. This type of operation is called a thyroidectomy.

In the neck, on either side of the thyroid gland, there are tiny glands called the parathyroid glands. Each one is approximately the size of a grain of rice. The parathyroid glands are responsible for the control of calcium levels in the blood. A common complication of a thyroid operation is accidentally damaging or removing the parathyroid glands. This can result in a condition called ‘hypoparathyroidism’. This condition can be temporary but in some cases it can be a long term condition, requiring lifelong medication and care.

A new technique using fluorescence (Glow) has been developed to help surgeons identify the parathyroid glands during surgery and reduce the risk of damage to these glands. This technology is called Near-Infrared Fluorescence (NIRF) Imaging.

We want to know if using this new technology (NIRF Imaging) during thyroid operations will reduce the number of patients who have parathyroid damage after this type of surgery, and so we are running this study to find out.

Why have I been chosen?

You have been asked to take part in the NIFTy study because you have been diagnosed with a condition of the thyroid and require an operation to remove your thyroid gland.

Do I have to take part?

No, your participation in the NIFTy study is voluntary.

If you decide to take part, you will be given this information document to keep and you will be asked to sign a consent form. Even if you agree to take part, you are free to withdraw at any time and without giving a reason.

If you decide not to take part, you will still receive the operation that you need. Your treatment and care will not be affected in any way.

If I want to, will I definitely be able to take part?

Unfortunately, no. Although your doctor thinks you might be suitable to take part, they will still need to carry out some tests and ask you some questions to make sure you are suitable. If it is not appropriate for you to take part in NIFTY, you will proceed with the standard surgical treatment that your doctor has discussed with you.

What will happen to me if I take part?

**Randomisation:**

The best way to find out whether the new treatment (surgery with NIRF imaging) is better than standard treatment (surgery without NIRF imaging) is in a randomised study. ‘Randomised’ means that a computer will allocate you randomly (as if by the roll of dice) to receive surgery with NIRF imaging or surgery without NIRF imaging. Neither your doctor nor you will choose which treatment you receive. In this way, a fair comparison can be made. Half the participants who take part in the study will receive the new treatment, and half will receive the standard treatment.

You may be asked some questions about your medical history to see if you are suitable for NIFTy. Before your operation you will have the usual routine blood tests but you will not need any other tests to take part in this trial.

You will receive your operation either with or without NIRF imaging depending on the randomisation allocation. The study does not influence the nature or extent of the operation. It will only affect how the operation will be carried out (i.e. with or without NIRF imaging).

**Follow up and Questionnaires:**

You will be reviewed at the hospital 1 day after your operation and will be asked to come in to hospital for clinic visits at around 1 month and 6 months following your operation when study data will be collected from you.

You will also be asked to fill in questionnaire booklets about your quality of life and how you are feeling. We will ask you to complete these booklets prior to trial entry (baseline) and at 1 month and 6 months after your operation. Some of these questionnaires will be given to you in clinic, whilst others will be posted directly to you, or you will have the option to complete the questionnaires online. These might take around 15 to 20 minutes of your time in each case.

What is the standard treatment?

The standard treatment for conditions of the thyroid such as yours is to have an operation called a thyroidectomy. You may need to have a ‘total thyroidectomy’ where all of your thyroid gland is removed or a ‘completion thyroidectomy’ where you will have had a previous partial thyroid removal and remaining thyroid tissue needs to be removed. Your surgeon will discuss the type of operation you require with you.

What is the new treatment?

The new treatment involves the same operation as the current standard treatment with the addition of Near Infrared Fluorescence Imaging (NIRF). During the operation a special infrared camera will be used to detect the natural glow of the parathyroid glands under infrared light. Then a dye (Indocyanine green, also called ICG) will be injected into your blood. The infrared camera will detect glow from the dye showing the blood supply to the parathyroid glands. The glow from parathyroid glands and the dye help to show up the glands and their blood supply more clearly.

The technology is registered (CE marked) for use in thyroid and parathyroid surgery and ICG has been in clinical use for many years for evaluation of blood flow.

Thyroidectomy details

The surgery will be under general anaesthesia, and will usually take around 2-3 hours (Use of the NIRF imaging may prolong the operation by around 15 minutes). After the surgery you will stay in hospital for 1-2 days, depending on your recovery and other medical problems you may have. You will be prescribed thyroxine hormone treatment to replace the function of the thyroid gland that has been removed.

Unwanted effects of treatment

There are known complications associated with any operation and your surgeon will discuss these with you. Your operation would take place whether or not you take part in this trial therefore these are the same risks as standard care.

Patients randomised to surgery with NIRF imaging will receive up to a maximum of 6 doses of Indocyanine Green (ICG). ICG has been in clinical use for many years for evaluation of blood flow. Expected complications related to the administration of ICG include severe allergic reactions, however this occurs very rarely and less than 1 in every 10,000 times the drug is used. There are a few circumstances when the ICG drug has to be used with caution, such as in patients who are taking certain medications. Your surgeon will go through these precautions in detail with you before the operation.

**Pregnancy:** It is important to tell your clinical care team if you are pregnant or become pregnant as this may affect your care.

How is my condition monitored?

Following a thyroidectomy you will be regularly monitored by your health care team on the hospital ward until you are discharged. Participating in the NIFTy study will not affect your long term care.

Clinical data about your condition and any complications that occur will be collected for the purposes of the study from the time of the treatment up to 6 months after your operation. The study follow-up schedule requires you to attend the hospital 1 month and 6 months after your operation but these visits should coincide with normal clinical care. The questionnaires you will be asked to complete 1 month and 6 months after your operation are not part of routine clinical care and are completed for the purpose of the study.

What are the possible disadvantages and risks of taking part?

Your clinician has recommended that you have an operation due to your thyroid condition. You will receive this operation whether or not you take part in this study. The risks from this operation are largely the same inside or outside of the study. There is a slight additional risk for patients who are randomised to the surgery with NIRF imaging arm, due to the administration of the ICG. Should you take part in the study you will need to spend some time completing questionnaires and it will be necessary for you to attend hospital appointments at certain times, but where possible these will be timed to coincide with normal clinical care.

What are the possible benefits of taking part?

We don’t know whether there will be a benefit to you. NIRF imaging may or may not improve thyroid surgery and reduce the rates of post-surgical hypoparathyroidism. The information gained from the study will help guide clinicians as to the best approach for thyroid surgeries in the future which will benefit other sufferers of thyroid conditions. You will be monitored regularly and closely throughout the study.

What if something goes wrong?

As with any surgical treatment, your clinical team aim to ensure that any risks are kept to a minimum. We have no reason to believe that surgery with NIRF imaging is less effective than standard surgery without NIRF but we cannot be 100% sure that this is the case. The Trial Management Group and the independent oversight committees will closely monitor the study on an on-going basis so that if there are any problems, they will be detected as soon as possible so that the study can be changed or stopped if necessary. If you experience problems, you must report these to your study nurse or doctor.

What happens when the research study stops?

At the end of the study you will carry on with normal clinical care as guided by your hospital.

Will my taking part be kept confidential?

If you decide to participate in the NIFTy study the information collected about you will be handled confidentially and strictly in accordance with relevant data protection laws, including the Data Protection Act 2018. Please refer to Part 2 and Part 3 for further details.

In line with Good Clinical Practice guidelines, at the end of the study, your data will be securely archived for a minimum of 15 years, after which arrangements for confidential destruction will be made.

**Contact Details**

If you have any further questions about your illness or clinical studies, please talk to your doctor or nurse. If you would like further information about clinical research, the UK Clinical Research Collaboration (a partnership of organisations working together on clinical research in the UK) have published various resources to help people learn more about clinical trials. Contact UKCRC: Tel: 0207 395 2271; email: [info@ukcrc.org](mailto:info@ukcrc.org); website [www.ukcrc.org](http://www.ukcrc.org).

**This completes Part 1 of the Information Sheet. If the Information in Part 1 has interested you and you are considering participation, please continue to read the additional information in Parts 2 and 3 before making any decision.**

**Part 2 – more about this study**

What if relevant new information becomes available?

Sometimes during the course of a study, new information becomes available for example, new surgical techniques may become available. If this happens, your surgeon will discuss with you how this affects you. You will be free to withdraw from the study at any time

What will happen if I don’t want to carry on with the study?

You can stop taking part in all of this study, or in any part of it, at any time and without giving a reason. However, we would like to know the reason if you are willing to say. Before deciding to stop, you should talk to your study doctor or nurse. They can advise you and may be able to deal with any concerns you may have. If you decide to stop taking part at any time it will not affect the standard of care you receive.

If you decide to leave the study before your surgery you will receive the standard operation. Study visits and assessments can still go ahead, if you agree to this.

If you tell us that you want to stop completing quality of life questionnaires, we will stop asking you to complete them. You can still take part in the study if you stop these, and you can change your mind later and start completing them again, if you want.

If you decide to stop study visits or assessments, to make sure the research is still reliable, we will need to keep the information we have already collected about you, and include it in the study analysis. Unless you clearly tell us you don’t want us to, we will continue collecting information about your health from routine hospital visits, via your GP or through other contact between you and your hospital. This is to help ensure the results of the study are valid.

Who has organised, reviewed and funded the research and who will be supervising it?

The NIFTy study is being centrally co-ordinated by the Clinical Trials Research Unit (CTRU) at the University of Leeds. The study is funded by the National Institute for Health Research (NIHR) Efficacy and Mechanism Evaluation (EME) Programme, an MRC and NIHR partnership (project reference 17/11/27). To have obtained funding by the NIHR, the study had to go through review by experts who felt this study to be of relevance and importance to patients and NHS. An independent Data Monitoring & Ethics Committee (DMEC) and Trial Steering Committee (TSC) will be reviewing the study data on a regular basis to monitor the ethics, safety and progress of the study. This research has been reviewed and approved by an independent Research Ethics Committee (REC).

What if there is a problem?

We do not expect anything to go wrong, but if a medical emergency related to your treatment for this study occurs whilst you are at home, you should initially try to contact the hospital where you received your treatment. If this is not possible you should go to the Accident & Emergency (A&E) department at your local hospital. If you are unable to get to the hospital, you should contact the emergency on call GP.

Complaints

If you wish to complain, or have any concerns about any aspect of the way you have been approached or treated during the course of this study your doctor or nurse should be your first contact. The normal National Health Service (NHS) complaints mechanisms are also available and your clinical team will give you further information if necessary. The contact details of your local team can be found on page 1 of this information leaflet.

If you have any questions or concerns about how your information is being used in this study please refer to part 3 of this leaflet.

Harm

In the event that something does go wrong and you are harmed during the research and this is due to someone's negligence then you may have grounds for a legal action for compensation against Sheffield Teaching Hospital NHS Foundation Trust as the Sponsor Organisation, or the participating NHS Trust but you may have to pay your legal costs. The normal National Health Service complaints mechanisms will still be available to you. Any claims will be subject to UK law and must be brought in the UK.

If you have private medical insurance, you should tell your insurer that you are taking part in research. They will let you know if it affects your policy.

How will my information be used?

A member of the study team at your hospital will enter most of the information needed for the study directly into our secure database (held at the CTRU). The study team at your hospital will also send us some information by post. This will include your completed consent form.

You will be allocated a study number, which will be used along with your date of birth and initials to identify you on each electronic or paper form.

Sometimes we will also get information about you by email or fax. Emails will use your study identifying number and sometimes your initials and date of birth. Fax may be used to send us a copy of your completed consent form, or other trial forms if they need to reach us quickly.

Finally, some particularly sensitive documents may be sent to us via a ‘secure file transfer’. This means information is sent by the internet in a very secure way.

If you agree to take part in the study we would like to collect a copy of your completed consent form so we can check you have definitely agreed to take part. This means that people in the study team who are authorised to deal with consent forms will see your full name. These people are trained to treat your information with care, and the consent form will be stored securely at all times. This form will be sent to the CTRU separately from any other study forms.

If you choose to complete your study questionnaires on paper, your name and postal address will be collected, on a contact details form to enable the CTRU to send quality of life questionnaires to your home. If you choose to complete the questionnaires online, your mobile telephone number and/or email address will be collected on the contact details form so that the CTRU can send you the questionnaires electronically as well as text or email reminders. This form will be sent to CTRU by post, fax or secure electronic transfer. Even though we will protect your confidentiality at all times we do have a duty of care toward you. This means that if a researcher believes that you might be a danger to yourself (e.g. you are thinking about harming yourself) we are obliged to alert your local study team.

Every effort will be made to ensure that any further information about you that leaves the hospital will have information removed so that you cannot be recognised from it; this information will usually be removed by a member of the study team at your hospital, but may also be removed by the CTRU upon receipt.

Your data will be entered onto a secure database held at the CTRU.

Your healthcare records may be looked at by authorised individuals from the research team, the Sheffield Teaching Hospital Trust (the trial Sponsor) or the regulatory authorities to check that the study is being carried out correctly.

All the information we collect for this study will be made available to other researchers at the end of the study for additional research, including information you have already provided if you stop taking part in the study. However, information will only be shared for worthwhile research projects with appropriate ethical approvals, only in such a way that no individual people can be identified, and only when we are sure the other researchers will manage your data correctly and securely. Sharing of clinical trial data is an important way we can make the most of the time and effort required to carry out trials.

Unless you clearly tell us you don’t want us to, we will continue to obtain data about you if you stop study treatment or stop attending study visits. This is to help ensure the results of the study are valid.

Your data may be passed to other organisations (possibly in other countries where the data protection standards and laws are different to the UK) to monitor the safety of the treatment(s) that you are receiving; this data will have your name removed.

Involvement of the General Practitioner/Family Doctor (GP):

Your GP, and the other doctors involved in your healthcare, will be kept informed of your participation in this study.

Will any genetic tests be done?

No genetic tests will be done as part of this study.

What will happen to the results of the study?

When the study is complete the results will be published in a medical journal, but no individual participants will be identified. The results may also be summarised on the internet - these results will not identify any individual participants.

We will make sure you have a chance to find out the results of the study, if you would like them.

What will happen if I lose mental capacity during the trial period?

This is expected to be a very rare occurrence. It could happen to any patient whether or not they are a participant in this study, for example due to a complication of an operation (e.g. a significant stroke) or due to an entirely separate event (e.g. a head injury). If this did occur, no further study treatment would be given and data collection for the study would not continue. Information included up until this point will remain on file and will be included in the analysis.

**Part 3 – Extra information about data protection**

General information

Sheffield Teaching Hospital Trust (STHT), based in the United Kingdom is the sponsor for this study. The sponsor has delegated the responsibility of running the trial to the Clinical Trials Research Unit (CTRU) based at the University of Leeds (UoL). The term ‘we’ used in this section refers to both STHT and UoL. We will be using information from you and your medical records in order to undertake this study and both organisations (STHT and University of Leeds) will act as joint data controllers for this study. This means that we are both responsible for looking after your information and using it properly. Data collected will be stored at the University of Leeds who will keep identifiable information about you for at least 15 years after the study has finished.

Your rights to access, change or move your information are limited, as we need to manage your information in specific ways in order for the research to be reliable and accurate. If you withdraw from the study, we will keep the information about you that we have already obtained. To safeguard your rights, we will use the minimum personally-identifiable information possible.

You can find out more about how your information is used at <https://ctru.leeds.ac.uk/privacy/>

Universities use personally-identifiable information to conduct research to improve health, care and services. As a publicly-funded organisation, we have to ensure that it is in the public interest when we use personally-identifiable information from people who have agreed to take part in research. This means that when you agree to take part in a research study, we will use your data in the ways needed to conduct and analyse the research study.

Health and care research should serve the public interest, which means that we have to demonstrate that our research serves the interests of society as a whole. We do this by following the UK Policy Framework for Health and Social Care Research.

If you wish to raise a complaint on how we have handled your personal data, you can contact the University of Leeds Data Protection Officer who will investigate the matter.

You can do this using any of the details below. If you do contact them, please mention the name of this study (NIFTy) and the Clinical Trials Research Unit.

- Email: [DPO@leeds.ac.uk](mailto:DPO@leeds.ac.uk)
- General postal address: University of Leeds, Leeds LS2 9JT, UK
- Postal address for data protection issues: University of Leeds, Room 11.72 EC Stoner Building, Leeds, LS2 9JT
- Telephone number: +44 (0)113 243 1751

If you are not satisfied with the response or believe your personal data has been processed in a way that is not lawful you can complain to the Information Commissioner’s Office (ICO).

- General website: ico.org.uk
- ICO contact webpage: ico.org.uk/global/contact-us
- Telephone number: 0303 123 1113
- Postal address: Information Commissioner’s Office, Wycliffe House, Water Lane, Wilmslow, Cheshire, SK9 5AF

How your information is used

Your hospital will collect information from you and/or your medical records for this research study in accordance with our instructions.

Your hospital will use your name, NHS number and contact details to contact you about the study, and make sure that relevant information about the study is recorded for your care, and to oversee the quality of the study. Individuals from University of Leeds, Sheffield Teaching Hospitals Trust and regulatory organisations may look at your medical and research records to check the accuracy of the research study. Your hospital will pass these details to the University of Leeds along with the information collected from you and your medical records. The only people in the University of Leeds who will access information that identifies you will be people who need to contact you in order to send questionnaires to you or audit the data collection process.

The University of Leeds will collect information about you for this research study from your hospital. This information will include your name, NHS number, contact details, initials, date of birth, study ID number and health information, which is regarded as a special category of information. We will use this information to help answer the research question. We need your contact details so that we can send you the Quality of Life questionnaires and reminders.

Your hospital will keep identifiable information about you from this study for at least 15 years after the study has finished.

Using your information for further research

When you agree to take part in a research study, the information about your health and care may be provided to researchers running other research studies in this organisation and in other organisations. These organisations may be universities, NHS organisations or companies involved in health and care research in this country or abroad. Your information will only be used by organisations and researchers to conduct research in accordance with the UK Policy Framework for Health and Social Care Research (<https://www.hra.nhs.uk/planning-and-improving-research/policies-standards-legislation/uk-policy-framework-health-social-care-research/>).

This information will not identify you and will not be combined with other information in a way that could identify you. The information will only be used for the purpose of health and care research, and cannot be used to contact you or to affect your care. It will not be used to make decisions about future services available to you, such as insurance.

**Delete this line, then print on Trust/Hospital headed paper**

| Participant ID: | Initials: |
| --- | --- |
| Date of Birth: | NHS Number: |
| ISRCTN: | Principal Investigator: |

**
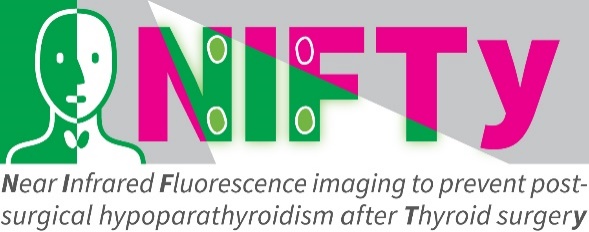
**

**PARTICIPANT CONSENT FORM**

***Please initial each box***

1. I confirm that I have read and understand the information sheet for the above study and have had the opportunity to ask questions.
2. I understand that my participation in this study is voluntary and that I am free to withdraw at any time without my medical care or legal rights being affected. I understand that even if I withdraw from the above study, the data collected from me will be used in analysing the results of the study.
3. I understand that my healthcare records may be looked at by authorised individuals from the study team, regulatory bodies or Sponsor in order to check that the study is being carried out correctly.
4. I agree to complete the Quality of Life questionnaires and understand that my full name and address, or my email address and/or telephone number if I choose to complete the questionnaires online, will be passed to the CTRU for the sole purpose of issuing these questionnaires.
5. I understand that if during this study my clinical care team determine that I have lost my ability to make my own decisions, no further study intervention will be given. I agree that data collected up until this point will remain on file and will be included in the analysis.
6. I understand that information or results arising from this study may be used for healthcare and/or further medical research and understand that my identity will remain anonymous wherever possible.
7. I understand that data may be passed to other organisations participating in this study (possibly in other countries where the data protection standards and laws are different to the UK) to monitor the safety of the treatment I am receiving.
8. I agree to a copy of this Consent Form being sent to the CTRU.
9. I agree that my GP, or any other doctor treating me, will be notified of my participation in this study.
10. I agree to take part in the study.

**Patient:**

Signature…………………………………………………………………………………

Name (block capitals)……………………………………………….……………………

Date………………………………………………….……………………………………

**Investigator:**

I have explained the study to the above named patient and they have indicated their willingness to participate.

Signature…………………………………………..……………………………………

Name (block capitals)……………………………………………….…………………

Date………………………………………………….……………………………………

**(If used)Translator:**

Signature…………………………………………..……………………………………

Name (block capitals)……………………………………………….…………………

Date………………………………………………….……………………………………

(1 copy for patient; 1 for the CTRU; 1 held in patient notes, original stored in Investigator Site File)
