# Supplementary material for: NIFTy: near-infrared fluorescence (NIRF) imaging to prevent postsurgical hypoparathyroidism (PoSH) after thyroid surgery—a phase II/III pragmatic, multicentre randomised controlled trial protocol in patients undergoing a total or completion thyroidectomy
Source: BMJ Open. 2025 Jan 30;15(1):e092422. doi: 10.1136/bmjopen-2024-092422 (PMC11784174; doi:10.1136/bmjopen-2024-092422)
Supplement: Supplementary file 5 [file bmjopen-2024-092422.reviewer_comments.pdf]

## PEER REVIEW HISTORY

BMJ Open publishes all reviews undertaken for accepted manuscripts. Reviewers are asked to complete a checklist review form and are provided with free text boxes to elaborate on their assessment. These free text comments are reproduced below.

### ARTICLE DETAILS

### Title (Provisional)

NIFTy: Near Infrared Fluorescence (NIRF) Imaging to prevent Post-surgical Hypoparathyroidism (PoSH) after Thyroid Surgery - A phase II/III pragmatic, multicentre randomised controlled trial protocol in patients undergoing a total or completion thyroidectomy

### Authors

Croft, Julie; Ainsworth, Gemma; Corrigan, Neil; Gordon, Katie; Perry, Anna; Twiddy, Maureen; Strachan, Mark; Wadsley, Jonathan; Mehanna, Hisham; Sharma, Neil; Glenister, Elizabeth; Stocken, Deborah D; balasubramanian, sabapathy prakash P

---

This article was not externally reviewed at BMJ Open. Protocols that have been independently assessed prior to submission to BMJ Open are usually fast-tracked to publication on the grounds that further substantial changes will not be possible. This independent assessment will usually be external, independent review for both a substantial grant award from a non-commercial or government funder and ethics approval.
